# The Inventory of Depressive Symptomatology Self Report (IDS-SR): Psychometric properties of the Indonesian version

**DOI:** 10.1371/journal.pone.0187009

**Published:** 2017-10-23

**Authors:** Retha Arjadi, Maaike H. Nauta, Dharmayati B. Utoyo, Claudi L. H. Bockting

**Affiliations:** 1 Department of Clinical Psychology and Experimental Psychopathology, University of Groningen, Groningen, The Netherlands; 2 Faculty of Psychology, Atma Jaya Catholic University of Indonesia, Jakarta, Indonesia; 3 Faculty of Psychology, University of Indonesia, Depok, Indonesia; 4 Department of Psychiatry, Academic Medical Center, University of Amsterdam, Amsterdam, The Netherlands; Universita Cattolica del Sacro Cuore Sede di Roma, ITALY

## Abstract

**Background:**

Depression screening and examination in Indonesia are highly challenging due to the disproportionately low number of mental health professionals in comparison to the Indonesian population. Self-report questionnaires on depression are cost-effective and time-efficient. The current study investigates the psychometric properties of the Indonesian Inventory of Depressive Symptomatology Self Report (IDS-SR).

**Methods:**

The participants were 904 Indonesians (aged 16–61; 50.2% female), recruited via an online survey using Qualtrics. Confirmatory factor analysis of the one-factor, three-factor, and four-factor model were explored. Convergent and divergent validity of the total score of the Indonesian IDS-SR and each factor were examined, as well as the Cronbach’s Alpha reliability. In addition, an optimal cut-off score for the Indonesian IDS-SR was established using ROC curve analysis.

**Results:**

The three-factor model of “cognitive/mood”, “anxiety/arousal”, and “sleep disturbance” was the best fit with the Indonesian IDS-SR data. Convergent and divergent validity were good. Cronbach’s Alpha reliability was excellent for the total score, good for the factors “cognitive/mood” and “anxiety/arousal”, but insufficient for the factor “sleep disturbance”. The optimal cut-off score of the Indonesian IDS-SR was 14, with 87% sensitivity and 86% specificity.

**Conclusions:**

As a multifactorial instrument to measure depression that has good validity and reliability, the Indonesian IDS-SR can be used to assess depressive symptoms for the purpose of research and clinical practice. The optimal cut-off score of the Indonesian IDS-SR is in accordance with the internationally used cut-off score.

## Introduction

As one of the most common mental health disorders, depression affects people around the world, including Indonesians. A recent investigation showed that the prevalence of depression in Indonesia is approximately 5% [1], but there are less than 5 mental health professionals per 100.000 people [2]. In Indonesia, it is unlikely that every depressive disorder is formally diagnosed by a trained professional based on a structured clinical interview due to a highly limited human resources, time, and money.

Self-report assessment is useful in detecting depression, and it is cost-effective and time-efficient. Self-reports have been shown to be able to identify depression with some degree of confidence [3]. Therefore, the availability of a good quality depression self-report measure can be beneficial under the challenging conditions in Indonesia. However, we are not aware of any freely available Indonesian depression self-report assessment with published psychometric properties that are satisfactory.

There are a number of depression self-report assessments that have been developed worldwide, and are frequently being used in clinical practice and research. The Inventory of Depressive Symptomatology Self Report or IDS-SR [4–5] is one example of a freely available depression measurement tool with good psychometric properties. It has been used widely in many studies in clinical populations [e.g. 6–7] as well as in community populations [e.g. 8–9]. The IDS-SR also has been translated and cross-validated into 30 languages. See www.ids-qids.org for all available translated instruments.

To be able to use the IDS-SR in Indonesia, it is first important to check whether the factor structure is comparable to the IDS-SR in other countries. Three different factor models have been proposed for the IDS-SR, namely a one-factor model [10], a three-factor model [5], and a four-factor model [4]. Next, it is necessary to investigate its reliability and validity, and also to estimate its optimal cut-off score. Therefore, the current study aims to investigate the factor structure, validity, reliability, and optimal cut-off score of the Indonesian IDS-SR.

## Materials and methods

### Subjects

The participants were Indonesians age 16 years old and above. There were 1622 individuals viewed the first page of the survey on Qualtrics, and 904 participants (55.7%) completed the survey. The participants’ age ranged from 16–61 years (*M* = 27.07, *SD* = 7.06), with 454 females (50.2%).

The sample came from different ethnic groups: 39.2% participants identified themselves as Javanese, 11.8% as Sundanese, 4% as Minangkabau ethnic group, 3.8% as Batakese, and the rest 41.2% were from at least 26 other ethnicity backgrounds. Among the participants, 72% were single, 26.5% were married, 1.5% were divorced/separated/widowed. The participants’ education level were below senior high (2.6%), senior high (29.4%), vocational degree (8.6%), bachelor degree (52.7%), and master degree (6.7%). The occupations of the participants were students and college students (31.4%), private and civil employees (31.8%), entrepreneurs (15.7%), and others (21.1%). Most participants lived in Jakarta (32%), Bandung (9.4%), Surabaya (7%), and Yogyakarta (6.4%), while the rest (45.2%) lived in other parts of Indonesia.

Based on the cut-off score of 6 as an indication of being depressed on the Indonesian version of the Beck Depression Inventory (BDI) [11], total 46.9% participants were categorized as not depressed and 53.1% were categorized as depressed.

### Main measure instrument

#### Inventory of Depressive Symptomatology Self Report (IDS-SR)

The IDS-SR [4–5] is a 30-item questionnaire measuring depressive symptoms. Each item has four statements that reflect various degrees of symptom severity, scored on a four-point scale from 0 to 3. There are two items about either increase or decrease in appetite, and two items about either increase or decrease in weight. Only the item with the higher score from both pairs was chosen. The total score is based on 28 items and ranges from 0 to 84.

The original version of the IDS-SR was translated into Indonesian language and then back translated by two independent translators to ensure the translation correctness. Differences between the original version and the back-translated version were discussed with a bilingual clinical psychologist from Indonesia in order to check the expressions according to Indonesian culture.

### Instruments for validation

#### Beck Depression Inventory (BDI, Indonesian version)

The Indonesian BDI [11] was adapted from the original BDI [12]. The scale has 21 items. Each item has four to six statements reflecting different degrees of symptom severity, and scored from 0 to 3 depending on the severity. The cut-off score of being depressed for the Indonesian BDI is 6. The reliability as reported by Cronbach’s Alpha coefficient of this measure in this study was high (*α* = 0.94).

#### Positive and Negative Affect Scale (PANAS)

The PANAS [13] consists of 10 items that measure positive affects and 10 items that measure negative affects. It has a five-point Likert scale from 1 (very slightly or not at all) to 5 (extremely). The internal consistency was high, *α* = 0.89 for PANAS positive items and *α* = 0.91 for PANAS negative items.

#### Subjective Happiness Scale (SHS)

The SHS [14] is a 4-item measure of subjective happiness with seven-point Likert scale. Responses from all items were summed and divided by four to provide a single composite score, ranging from 1 to 7. The SHS reliability was good (*α* = 0.79).

### Procedure

This study was part of a longer survey about the acceptability of online interventions for depression in Indonesia. The whole survey was conducted as a first step in the process of developing an internet-based intervention for depression in Indonesia and testing its effectiveness afterwards in a clinical trial. The output of the current study (the Indonesian IDS-SR) will be used as one of the assessment tools in the clinical trial.

The data collection of this study was conducted through an internet-based platform for surveys (www.qualtrics.com). The recruitment of survey participants was done via invitations on our own study website (www.actandfeel.com), as well as via two other websites focused on mental health issues, online forums about mental health on social media, general social media, and by word of mouth. Consent statements were provided at the beginning of the online survey page and participants could tick an agree button to indicate their agreement. The first author provided her contact information in case the participants had any concerns regarding the study.

This study was approved by Tarumanagara University Human Research Ethics Committee, Indonesia, under project number PPZ20142001. The ethics committee approved the inclusion of participants <18 years old (16–17 years old) without parent/guardian consent.

### Data analyses

A confirmatory factor analysis (CFA) was used to test the factor structure of the Indonesian IDS-SR according to the one-factor model of “depression” [10], three-factor model of “cognitive/mood”, “anxiety/arousal”, and “sleep disturbance” [5], and four-factor model of “mood/cognition”, “anxious/hypochondriacal”, “endogenous”, and “atypical” [4]. Some items loaded in more than one factor in the original findings of three-factor and four-factor model. In that case, we placed them in the factor of which they had the highest loading coefficient, to ensure that one item only referred to a single factor. The fit of each model was assessed by the following fit indices: *CFI* (Comparative Fit Index) and *RMSEA* (Root Mean Square Error of Approximation) as noncentrality-based indices, and *SRMR* (Standardized Root Mean Square Residual) and *AIC* (Akaike’s Information Criterion) as absolute fit indices. The rule-of-thumb guidelines to indicate a good fit were defined as: *CFI*>0.90, *RMSEA*<0.05, *SRMR*<0.05 [15]. It is not possible to directly compare the fit indices of each model if they are not nested on each other. We used the *AIC* to compare non-nested models, with lower *AIC* indicating a better fit [16]. The CFA was calculated using *R* (ver. 3.1.1) with the Lavaan package ver. 0.5–17 [17]. The CFA was estimated through a maximum likelihood procedure with a robust standard error and Satorra-Bentler correction. The CFA model was then plotted with the semPlot package ver 1.0.1 [18].

The reliability coefficients of the whole scale and of singular factors were calculated. Bivariate Pearson correlation were used to calculate the convergent and divergent validity of the Indonesian IDS-SR. Convergent validity was calculated by correlating the Indonesian IDS-SR to the Indonesian BDI and the positive affect scale of the PANAS. Divergent validity was assessed by correlating the Indonesian IDS-SR to the negative affect scale of the PANAS and the SHS. Finally, we also conducted a Receiver Operating Characteristic (ROC) curve analysis to test determine the optimal cut-off point of the Indonesian IDS-SR based on the cut-off point of 6 on the Indonesian BDI as an indication of being depressed. The reliability, convergent and divergent validity analyses, and cut-off determination was calculated using IBM SPSS Statistics 22. The anonymized dataset is made publicly available in an online repository.

## Results

### Confirmatory factor analysis

**Fig 1:**
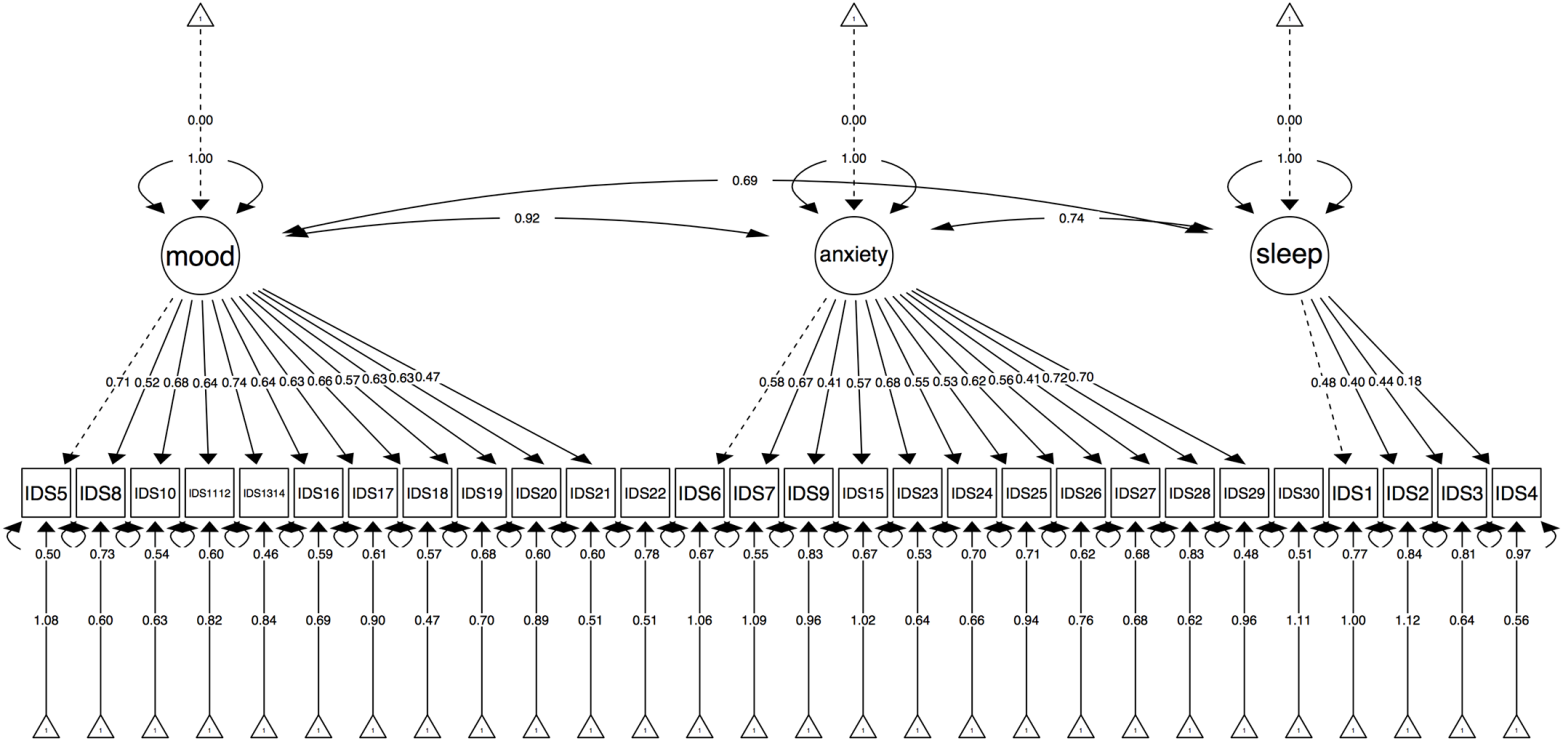
Three-factor model with standardized factor loading of the Indonesian IDS-SR.

The fit indices showed that the one-factor and three-factor model possibly fit with the data, but the three-factor model fitted best. The CFA results for the four-factor model could not be interpreted because the covariance matrix was not positive definite: the latent variable “mood/cognition” was linearly dependent on the latent variable “endogenous” (correlation above one). The one-factor model met the sufficient value of *RMSEA* and *SRMR*, but not the *CFI* (*RMSEA* = 0.045, 95% *CI* = 0.042–0.048; *SRMR* = 0.041; *CFI* = 0.894). The completely standardized factor loadings ranged from 0.18 to 0.73, with a mean of 0.55. Meanwhile, the three-factor model met rule-of-thumbs criteria of all fit indices: *CFI* = 0.910, *RMSEA* = 0.041, 95%*CI* = 0.039–0.044, and *SRMR* = 0.039. When the models were compared, the three-factor model had the best fit with lowest *AIC* (52958.64) compared to the one-factor model (53082.95). See Table 1 for the descriptive information of the Indonesian IDS-SR total score and each factor score from the three-factor model. Fig 1 presents factors loadings for the three-factor model of the Indonesian IDS-SR.

**Table 1:** Descriptive of the Indonesian IDS-SR three-factor model.

	N = 904	
	Range	M (SD)
**IDS-SR Total**	0–74	18.42 (13)
**Factor 1: Mood**	0–34	7.27 (6.72)
**Factor 2: Anxiety**	0–32	7.92 (5.72)
**Factor 3: Sleep**	0–10	3.23 (2.24)

### Reliability

The reliability of the Indonesian IDS-SR was *α* = 0.92. Removal of any of the Indonesian IDS-SR items did not lead to a significant increase of any reliability coefficient, reflecting satisfactory scale homogeneity of the total scale. The reliability coefficients for the separate factors were: *α* = 0.88 (Mood, 12 items), *α* = 0.86 (Anxiety, 12 items), and *α* = 0.38 (Sleep, 4 items).

### Convergent and divergent validity

The convergent validity of the Indonesian IDS-SR and each of its three factors were shown by the high positive correlation with the Indonesian BDI and the negative affect scale of the PANAS. The divergent validity was shown by the negative correlation with the positive affect scale of the PANAS and the SHS. The correlations matrix is presented in Table 2.

**Table 2:** Correlations.

	**Indonesian IDS-SR total**	**Indonesian IDS-SR factors**			**Indonesian BDI**	**PANAS** **(Negative)**	**PANAS** **(Positive)**	**SHS**
		**Mood**	**Anxiety**	**Sleep**				
**Indonesian IDS-SR total**	1	0.94*	0.93*	0.60*	0.89*	0.69*	-0.51*	-0.64*
**Factor 1: Mood**	-	1	0.80*	0.43*	0.90*	0.64*	-0.58*	-0.67*
**Factor 2: Anxiety**	-	-	1	0.46*	0.80*	0.69*	-0.40*	-0.54*
**Factor 3: Sleep**	-	-	-	1	0.43*	0.35*	-0.19*	-0.29*
**Indonesian BDI**	-	-	-	-	1	0.66*	-0.56*	-0.66*
**PANAS (Negative)**	-	-	-	-	-	1	-0.34*	-0.58*
**PANAS (Positive)**	-	-	-	-	-	-	1	0.65*
**SHS**	-	-	-	-	-	-	-	1

* All correlations were significant at *p* < .05 (two-tailed)

### Optimal cut-off score

**Fig 2:**
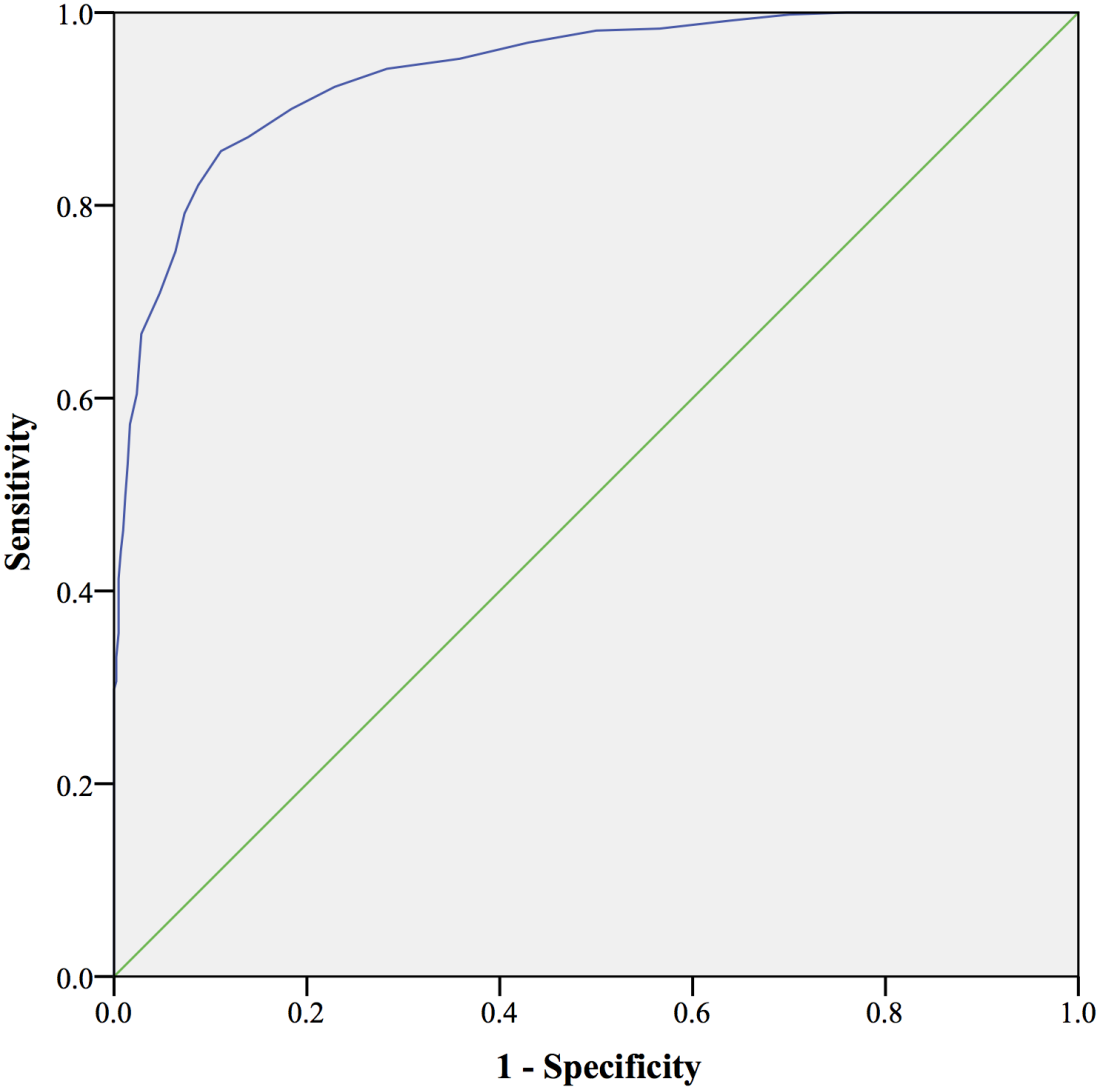
ROC curve of the Indonesian IDS-SR.

The ROC curve analysis using the Indonesian BDI cut-off score (≥6) as the state variable showed that the area under ROC curve (AUC) for the Indonesian IDS-SR was 0.939 (*CI*: 0.925–0.954, *p* = .000), indicating a high classification accuracy [19–20] (See Fig 2). It means the Indonesian IDS-SR was able to differentiate between those who were and were not having a depression. Furthermore, the optimal cut-off score is attained when the weight of sensitivity and specificity is equal [21]. According to this standard, in our sample, the best diagnostic accuracy for the Indonesian IDS-SR was 14, identical with the internationally used cut-off for mild depression [22]. The sensitivity and the specificity were 87% and 86% respectively. See Table 3.

**Table 3:** Sensitivity and specificity values of the Indonesian IDS-SR at different cut-off scores.

Cut-off score	Sensitivity (%)	Specificity (%)
6	99	36
7	98	43
8	98	50
9	97	57
10	95	64
11	94	72
12	92	77
13	90	82
14	87	86
15	86	89
16	82	91
17	79	93
18	75	94
19	71	95
20	67	97
21	60	98
22	57	98
23	53	99
24	50	99

## Discussion

The results of the study indicate that the Indonesian IDS-SR has good psychometric properties, similar to previous studies in the Netherlands and China [23–24]. Both the one-factor model and the three-factor model had a good fit with the data, with indications for a better fit of the three-factor solution, also in line with previous findings that compared several factorial structures of the IDS-SR in other countries [23,25]. The reliability, convergent and divergent validity were excellent for the total scale and for the “cognitive/mood” and “anxiety/arousal” factors. The “sleep disturbance” factor, however, had a relatively low convergent and divergent validity, as well as low reliability. These results are in line with previous studies indicating that the “sleep” factor may not be sufficient to be used as a subscale. The various sleep problems seem to be quite heterogenic, and the scale does not reflect one single construct [5,23,26]. Nevertheless, the three factors altogether are good to measure symptoms of depression.

This study has some limitations. First, based on the Indonesian BDI cut-off score, it can be seen that half of our participants were categorized as depressed. Our current sample may have included more depressed, highly educated, and young participants than a general population due to the recruitment strategy that fully online and partly took place through websites and online forum on mental health, which may have attracted people with (sub)clinical complaints. Therefore, we recommend future studies to replicate this study in a more representative sample from the Indonesian population and compare scores from general population samples and pure clinical samples.

The second limitation is that we used another self-report, which is the Indonesian BDI, as a golden standard to determine an optimal cut-off of the Indonesian IDS-SR. To reconfirm the results, future studies may include a structured clinical interview (e.g. SCID-5 [27]) as a standard on the diagnosis of depressed and non-depressed participant, and reevaluate the currently proposed cut-off. Even though we regard our conclusions in this study as prudent based on the Indonesian BDI as the golden standard, we believe that using clinical interviews in future studies would be the best method to affirm the results. In addition, there was no information available on the Indonesian BDI regarding extensive different levels of depression severity (mild, moderate, severe, very severe) as used in the original IDS-SR, which made it not possible for us to do severity analysis on the Indonesian IDS-SR. Therefore, inclusion of a good severity measure for depression, such as the clinician-administered Hamilton Depression Rating Scale (HAM-D) [28] for future studies may also provide additional valuable information and enable further analysis on severity.

For the last limitation, in this study, we were not able to analyze responsiveness to change because we only had single-time data collection. Accordingly, we consider it is important for future research to collect multiple-time data point to allow further analysis on responsiveness to change.

## Conclusions

The current study showed that the commonly used three-factor model for the IDS-SR has the best fit for the Indonesian IDS-SR. This study also reported that the Indonesian IDS-SR has good validity, satisfactory reliability, and optimal cut-off score of 14, in line with the internationally used cut-off. Therefore, it can be concluded that the Indonesian IDS-SR is a valuable instrument for assessing depressive symptoms, both in clinical practice and research context.

## Supporting information

S1 File IDS-SR questionnaire (Bahasa Indonesia). (PDF)
