# Supplementary material for: The Inventory of Depressive Symptomatology Self Report (IDS-SR): Psychometric properties of the Indonesian version
Source: PLoS One. 2017 Oct 23;12(10):e0187009. doi: 10.1371/journal.pone.0187009 (PMC5653354; doi:10.1371/journal.pone.0187009)
Supplement: S1 File — (PDF) [file pone.0187009.s001.pdf]

Inventory of Depressive Symptomatology Self-Report (IDS-SR)
Bahasa Indonesia

Nama lengkap: ______________________________ **Tanggal:** ____________

Petunjuk: Pada setiap nomor, lingkarilah satu angka di samping jawaban yang paling menggambarkan kondisi Anda selama seminggu terakhir.

1. Tertidur
0 Saya tidak pernah membutuhkan waktu lebih dari 30 menit untuk tertidur.
1 Pada beberapa hari, saya membutuhkan waktu setidaknya 30 menit untuk tertidur (kurang dari 3 hari dalam seminggu terakhir).
2 Pada kebanyakan hari, saya membutuhkan waktu 30-60 menit untuk tertidur (lebih dari 3 hari dalam seminggu terakhir).
3 Pada kebanyakan hari, saya membutuhkan waktu lebih dari 60 menit untuk tertidur (lebih dari 3 hari dalam seminggu terakhir).

2. Tidur di malam hari
0 Saya tidak terbangun di malam hari.
1 Tidur saya resah, tidak nyenyak, dan saya terbangun singkat beberapa kali setiap malam.
2 Saya terbangun setidaknya satu kali dalam semalam, tetapi saya dapat kembali tidur dengan mudah.
3 Pada kebanyakan hari, saya terbangun lebih dari satu kali dalam semalam, dan tetap terjaga selama setidaknya 20 menit.

3. Bangun tidur terlalu awal
0 Pada kebanyakan hari, saya bangun kurang dari 30 menit sebelum waktu yang seharusnya.
1 Pada kebanyakan hari, saya bangun lebih dari 30 menit sebelum waktu yang seharusnya.
2 Saya hampir selalu bangun setidaknya 1 jam lebih awal dari waktu yang seharusnya, tetapi akhirnya saya dapat kembali tidur.
3 Saya selalu bangun setidaknya 1 jam lebih awal dari waktu yang seharusnya, dan tidak bisa kembali tidur.

4. Tidur berlebihan
0 Saya tidur maksimal 7-8 jam setiap malam, tanpa tidur siang.
1 Saya tidur maksimal 10 jam dalam 24 jam, termasuk tidur siang.
2 Saya tidur maksimal 12 jam dalam 24 jam, termasuk tidur siang.
3 Saya tidur lebih dari 12 jam dalam 24 jam, termasuk tidur siang.

5. Merasa sedih
0 Saya tidak merasa sedih.
1 Saya merasa sedih hanya pada beberapa hari.
2 Saya merasa sedih pada kebanyakan hari.
3 Saya merasa sedih hampir setiap saat.

6. Merasa mudah marah
0 Saya tidak merasa mudah marah.
1 Saya merasa mudah marah hanya pada beberapa hari.
2 Saya merasa mudah marah pada kebanyakan hari.
3 Saya merasa sangat mudah marah hampir setiap saat.

7. Merasa cemas atau tegang
0 Saya tidak merasa cemas atau tegang.
1 Saya merasa cemas atau tegang hanya pada beberapa hari.
2 Saya merasa cemas atau tegang pada kebanyakan hari.
3 Saya merasa sangat cemas atau tegang hampir setiap saat.

8. Respon suasana hati terhadap peristiwa yang menyenangkan
0 Ketika peristiwa menyenangkan terjadi, suasana hati saya menjadi cerah selama beberapa jam.
1 Ketika peristiwa menyenangkan terjadi, suasana hati saya menjadi cerah, tetapi saya tidak merasa seperti diri saya yang normal.
2 Hanya sebuah hal menyenangkan tertentu yang dapat membuat suasana hati saya menjadi sedikit lebih cerah.
3 Suasana hati saya sama sekali tidak menjadi cerah bahkan ketika ada hal-hal yang sangat menyenangkan terjadi dalam hidup saya.

9. Suasana hati terkait dengan waktu
0 Tidak ada hubungan antara suasana hati saya dengan waktu tertentu.
1 Suasana hati saya lebih berhubungan dengan situasi (cth: sedang sendirian, sedang bekerja) daripada dengan waktu tertentu.
2 Secara umum, suasana hati saya lebih berhubungan dengan waktu tertentu daripada dengan situasi tertentu.
3 Suasana hati saya jelas-jelas dapat diprediksi akan menjadi lebih baik atau lebih buruk pada waktu tertentu setiap harinya.

9A. Kapankah biasanya suasana hati Anda memburuk? (lingkari jika ada yang sesuai) Pagi hari / Siang hari / Malam hari
9B. Apakah perubahan pada suasana hati Anda ditentukan oleh situasi? (lingkari yang sesuai) Ya / Tidak

10. Sifat suasana hati
0 Suasana hati yang saya rasakan adalah suasana hati yang normal.
1 Suasana hati saya sedih, kurang lebih sama dengan rasa sedih yang akan saya rasakan jika orang dekat saya meninggal/pergi.
2 Suasana hati saya sedih, tetapi agak berbeda sifatnya dari rasa sedih yang akan saya rasakan jika orang dekat saya meninggal/pergi.
3 Suasana hati saya sedih, tetapi sangat berbeda dengan tipe kesedihan yang menyangkut perasaan berduka atau kehilangan.

Jawablah ANTARA nomor 11 atau 12 yang sesuai dengan kondisi Anda (tidak keduanya)

11. Penurunan selera makan
- 0 Saya tidak mengalami perubahan selera makan.
- 1 Saya makan agak lebih jarang atau lebih sedikit dari biasanya.
- 2 Saya makan jauh lebih sedikit dari biasanya dan hanya jika saya benar-benar mendorong diri untuk makan.
- 3 Saya jarang makan selama 24 jam, dan hanya akan makan jika benar-benar meniatkan diri atau jika ada yang membujuk.

12. Peningkatan selera makan
- 0 Saya tidak mengalami perubahan selera makan.
- 1 Saya merasa butuh lebih sering makan dari biasanya.
- 2 Saya lebih sering dan/atau lebih banyak makan dari biasanya.
- 3 Saya merasakan dorongan untuk makan berlebihan, baik saat jam makan maupun di luar jam makan.

Jawablah ANTARA nomor 13 atau 14 yang sesuai dengan kondisi Anda (tidak keduanya)

13. Berat badan turun
- 0 Saya tidak mengalami perubahan berat badan.
- 1 Saya merasa seolah berat badan saya sedikit turun.
- 2 Berat badan saya turun 1 sampai 2,5 kilogram.
- 3 Berat badan saya turun lebih dari 2,5 kilogram.

14. Berat badan naik
- 0 Saya tidak mengalami perubahan berat badan.
- 1 Saya merasa seolah berat badan saya sedikit naik.
- 2 Berat badan saya naik 1 sampai 2,5 kilogram.
- 3 Berat badan saya naik lebih dari 2,5 kilogram.

15. Konsentrasi atau pengambilan keputusan
- 0 Tidak ada perubahan dalam kemampuan saya untuk berkonsentrasi atau mengambil keputusan.
- 1 Adakalanya saya merasa ragu-ragu dalam memutuskan sesuatu atau mendapati diri saya sulit fokus.
- 2 Pada kebanyakan waktu, saya berjuang untuk dapat fokus atau untuk membuat keputusan.
- 3 Saya tidak bisa cukup berkonsentrasi untuk sekedar membaca, atau bahkan tidak bisa membuat keputusan-keputusan kecil.

16. Pandangan terhadap diri sendiri
- 0 Saya melihat diri saya sebagai orang yang sama berharganya dan sama layaknya dengan orang lain.
- 1 Saya lebih banyak menyalahkan diri dari biasanya atas berbagai ketidakberesan yang terjadi.
- 2 Saya yakin bahwa saya menyebabkan banyak masalah untuk orang lain.
- 3 Saya hampir selalu terus-menerus memikirkan berbagai kekurangan diri, kesalahan, dan masalah yang saya buat.

17. Pandangan terhadap masa depan diri
- 0 Saya memiliki pandangan yang optimis mengenai masa depan saya.
- 1 Adakalanya saya merasa pesimis mengenai masa depan saya, tetapi pada kebanyakan waktu saya yakin banyak hal akan membaik.
- 2 Saya cukup yakin bahwa tidak akan ada hal-hal baik yang menjanjikan untuk saya dalam waktu dekat (1-2 bulan ke depan).
- 3 Saya tidak melihat adanya harapan akan terjadinya hal baik apapun pada diri saya di masa depan.

18. Ide-ide mengenai kematian dan bunuh diri
- 0 Saya tidak berpikir mengenai bunuh diri atau kematian.
- 1 Saya merasa hidup ini kosong atau bertanya-tanya apakah hidup ini layak untuk dijalani.
- 2 Saya mendapati diri saya berpikir mengenai bunuh diri atau kematian beberapa kali dalam seminggu ini.
- 3 Saya terus berpikir secara detail mengenai bunuh diri atau kematian beberapa kali dalam sehari, atau sudah membuat rencana spesifik untuk bunuh diri, atau sudah benar-benar mencoba untuk mengakhiri hidup.

19. Minat umum
- 0 Tidak ada perubahan yang terjadi dalam minat saya untuk bergaul atau melakukan berbagai aktivitas.
- 1 Saya menyadari bahwa belakangan ini saya menjadi kurang berminat untuk bergaul atau melakukan berbagai aktivitas.
- 2 Saya mendapati bahwa saya hanya berminat terhadap satu atau dua aktivitas yang biasanya saya lakukan saja.
- 3 Saya sama sekali tidak berminat untuk melakukan berbagai aktivitas yang biasanya saya sukai.

20. Level energi
- 0 Level energi saya tidak berubah dari biasanya.
- 1 Saya menjadi lebih mudah lelah dari biasanya.
- 2 Saya harus berupaya keras untuk memulai atau menyelesaikan aktivitas harian yang biasa saya kerjakan (cth: belanja, pergi kerja).
- 3 Saya benar-benar tidak bisa melakukan sebagian besar aktivitas harian saya karena tidak punya energi untuk melakukannya.

21. Kapasitas untuk merasakan kesenangan dan kenikmatan (tidak termasuk aktivitas seksual)
- 0 Saya menikmati berbagai aktivitas yang santai/menyenangkan seperti biasanya.
- 1 Saya tidak merasakan kenikmatan yang biasanya saya rasakan ketika melakukan berbagai aktivitas yang santai/menyenangkan.
- 2 Saya jarang merasakan kesenangan dari aktivitas apapun.
- 3 Saya tidak mampu merasakan kesenangan atau kenikmatan dari apapun.

22. Minat seksual (pertanyaan berfokus hanya pada minat saja, bukan pada aktivitasnya)
- 0 Saya berminat pada seks seperti biasanya.
- 1 Minat saya terhadap seks agak menurun dari biasanya atau saya tidak memperoleh kesenangan dari seks seperti biasanya.
- 2 Saya memiliki sedikit hasrat untuk seks dan/atau hanya memperoleh sangat sedikit kesenangan dari seks.
- 3 Saya sama sekali tidak memiliki minat terhadap seks dan/atau sama sekali tidak memperoleh kesenangan dari seks.

23. Merasa lebih lambat dari biasanya
- 0 Saya berpikir, berbicara, dan bergerak pada kecepatan saya biasanya.
- 1 Saya mendapati kemampuan berpikir saya melambat dari biasanya atau suara saya terdengar jemu/datar.
- 2 Saya membutuhkan beberapa detik untuk merespon hampir semua pertanyaan dan saya yakin kemampuan berpikir saya melambat.
- 3 Saya seringkali tidak mampu merespon berbagai pertanyaan jika tidak betul-betul berusaha dengan keras.

24. Merasa gelisah

0 Saya tidak merasa gelisah.
1 Saya seringkali gelisah, meremas-remas tangan, atau harus bergerak-gerak ketika sedang duduk.
2 Saya merasa harus terus bergerak dan saya cukup gelisah.
3 Saya seringkali tidak bisa duduk diam dan harus mondar-mandir ke sana ke mari.

25. Sakit atau nyeri

0 Saya tidak merasakan sakit/nyeri apapun.
1 Kadang-kadang, saya mengalami sakit kepala atau nyeri pada perut, punggung, atau sendi, tetapi itu hanya muncul sesekali dan tidak sampai menghalangi saya beraktivitas.
2 Saya mengalami sakit/nyeri yang disebutkan di atas pada kebanyakan waktu.
3 Rasa sakit/nyeri yang saya alami begitu parah hingga memaksa saya untuk berhenti beraktivitas.

26. Gejala fisik lainnya

0 Saya tidak mengalami gejala-gejala berikut: jantung berdetak cepat, penglihatan kabur, berkeringat, panas dingin, nyeri dada, jantung berdebar, telinga berdenging, atau gemetaran.
1 Saya mengalami sedikit dari gejala yang disebutkan di atas, tetapi sifatnya ringan dan munculnya hanya kadang-kadang.
2 Saya mengalami beberapa gejala yang disebutkan di atas dan itu cukup mengganggu saya.
3 Saya mengalami beberapa gejala yang disebutkan di atas dan saat gejala-gejala itu muncul, saya menjadi harus berhenti beraktivitas.

27. Gejala panik atau phobia

0 Saya tidak mengalami serangan panik atau rasa takut/phobia yang spesifik (cth: terhadap binatang atau ketinggian).
1 Saya mengalami serangan panik atau rasa takut yang ringan, tetapi biasanya tidak sampai mempengaruhi perilaku saya atau membuat saya berhenti beraktivitas.
2 Saya mengalami serangan panik atau rasa takut yang nyata hingga mempengaruhi perilaku saya, tetapi <u>tidak</u> membuat saya berhenti beraktivitas.
3 Setidaknya satu kali dalam seminggu, saya mengalami serangan panik atau rasa takut yang parah hingga membuat saya berhenti melakukan aktivitas sehari-hari.

28. Konstipasi atau diare

0 Saya tidak mengalami perubahan dalam kebiasaan buang air besar.
1 Adakalanya saya mengalami konstipasi atau diare yang sifatnya ringan.
2 Saya mengalami konstipasi atau diare pada kebanyakan waktu, tetapi itu tidak mengganggu aktivitas saya sehari-hari.
3 Saya mengalami konstipasi atau diare yang sampai mengharuskan saya minum obat atau sampai mengganggu aktivitas harian saya.

29. Sensitivitas interpersonal

0 Saya sama sekali tidak mudah merasa ditolak, diremehkan, dikritik, atau disakiti oleh orang lain.
1 Adakalanya saya merasa ditolak, diremehkan, dikritik, atau disakiti oleh orang lain.
2 Saya seringkali merasa ditolak, diremehkan, dikritik, atau disakiti, tetapi perasaan tersebut hanya sedikit saja mempengaruhi hubungan saya dengan orang lain atau pekerjaan saya.
3 Saya seringkali merasa ditolak, diremehkan, dikritik, atau disakiti, dan perasaan tersebut sampai mengganggu hubungan saya dengan orang lain serta pekerjaan saya.

30. Tubuh terasa berat atau lemas

0 Tubuh saya tidak terasa berat atau lemas.
1 Adakalanya tubuh saya terasa berat dan lemas, namun tidak berefek negatif terhadap pekerjaan, studi, atau level aktivitas saya.
2 Tubuh saya terasa berat dan lemas pada kebanyakan hari.
3 Tubuh saya terasa berat dan lemas selama berjam-jam setiap hari.

<u>Petunjuk skoring:</u>

- Rentang skor: 0-84
- Jumlahkan total skor dari **28 item**. Perhatikan keterangan berikut:
  - *Pertanyaan nomor 9A dan 9B hanya untuk tambahan informasi, tidak termasuk dalam skoring*
  - *Gunakan satu skor antara jawaban nomor 11 atau 12 (jika keduanya diisi, pilih skor yang lebih tinggi)*
  - *Gunakan satu skor antara jawaban nomor 13 atau 14 (jika keduanya diisi, pilih skor yang lebih tinggi)*

TOTAL SKOR: ____

<u>If you use this instrument, please cite our article. / Jika Anda menggunakan alat tes ini, mohon kutip artikel kami</u>.
